# Ethics of Autonomous AI Clinical Trials: Delphi Study

**DOI:** 10.2196/91472

**Published:** 2026-08-21

**Authors:** Ariadne A Nichol, Alaa Youssef, David B Larson, Michael Abramoff, Risa M Wolf, Danton Char, Nicole Martinez-Martin

**Affiliations:** 1 Center for Biomedical Ethics, Stanford Medicine, 300 Pasteur Drive, Stanford, CA, 94305, United States, 1 (650) 723-4480; 2 Department of Radiology, Stanford Medicine, Stanford, CA, United States; 3 Department of Ophthalmology and Visual Sciences, University of Iowa Health Care, Iowa City, IA, United States; 4 Department of Pediatrics, Division of Endocrinology, Johns Hopkins Medicine, Baltimore, MD, United States; 5 Department of Anesthesiology, Division of Pediatric Cardiac Anesthesia, Stanford Medicine, Stanford, CA, United States; 6 Department of Psychiatry and Behavioral Sciences, Stanford Medicine, Stanford, CA, United States

**Keywords:** autonomous AI, clinical trial, ethics, Delphi, transparency, bioethics, public policy, consensus

## Abstract

**Background:**

Safe implementation of autonomous AI in medicine requires rigorous evaluation through clinical trials. The 7 guiding principles for ethical clinical research endorsed by the National Institutes of Health (NIH) provide an established framework for promoting scientific rigor and protecting the safety of human participants. However, clinical trials of autonomous AI raise novel ethical issues that require adaptation of these principles to account for effects that can vary across stakeholders and implementation contexts, including model performance across clinical settings. Incorporating expert perspectives on such challenges is critical to developing effective and ethically robust guidelines for autonomous AI clinical trials.

**Objective:**

This Delphi study aimed to generate expert consensus on how the National Institutes of Health’s 7 principles of ethical clinical research should be applied to clinical trials of autonomous AI.

**Methods:**

We conducted 2 rounds of surveys followed by a final virtual meeting using a modified Delphi approach with a multidisciplinary expert panel. Participants were purposively identified through PubMed literature searches and selected for expertise in AI, data science, ophthalmology, public policy, law, bioethics, and patient advocacy. Round 1 used open-ended survey questions based on a vignette describing an autonomous AI tool. Round 1 survey responses were analyzed qualitatively using thematic coding, and were used to generate representative statements for Round 2. In round 2, panelists rated statements on a 5-point Likert scale. In accordance with Delphi methodology, statements of consensus within the surveys (at least 80% rating agreement) and moderate agreement (60%‐80% rating agreement) were identified for further discussion and iteration. Findings from a final virtual meeting were then analyzed thematically and synthesized into actionable recommendations.

**Results:**

Fourteen expert panelists participated in the Delphi study over a 6-month period. Participation was 12 (85.7%) of 14 experts in round 1, 10 (71.4%) of 14 experts in round 2, and 13 (92.9%) of 14 experts in the final virtual meeting. Round 2 survey results yielded 9 strong agreement statements, 2 moderate agreement statements, and 4 divisive statements for participants to explore further in developing recommendations. Final recommendations from the virtual meeting addressed transparency regarding training and validation data, bias assessment before deployment, performance across clinical settings, health inequities before implementation, stakeholder engagement, informed consent, comparison of AI tools with existing standards of care, downstream access to care after AI-generated recommendations, and cost and access implications.

**Conclusions:**

Ethical evaluation of autonomous AI clinical trials should extend beyond technical accuracy to help mitigate potential harms to patients. This study highlights key ethical considerations for clinical trials of autonomous AI and provides consensus recommendations from a multidisciplinary Delphi panel. These recommendations can inform future research, policy, and guidance for the ethical development and implementation of autonomous AI clinical trials.

## Introduction

Autonomous AI is increasingly being evaluated for use in health care, raising important questions for the ethics of clinical research. Unlike AI-based decision support tools meant to assist clinicians, autonomous AI tools are designed to screen for disease or provide clinical recommendation outputs without specialist oversight at the point of care. As these tools move from development into clinical research trials, regulators must determine how established research ethics principles should apply to such novel technology.

This question has become more urgent, as AI-based medical devices have rapidly expanded. Since the first autonomous AI system received the US Food and Drug Administration (FDA) De Novo authorization in 2018, the number of AI medical devices authorized has grown rapidly, now exceeding 1000 devices [1]. Yet, clinical evaluation of AI tools in health care remains challenging [2-5]. Systematic reviews have highlighted significant limitations, including the lack of clinically relevant endpoints and a high risk of bias [6,7]. Harms from AI in health care, particularly bias and differing performance across populations, have often been discovered only after deployment [8-10]. Ethics principles for medical research provide guidance for assuring scientific benefit as well as protection of human subjects. There is a need for more explicit guidance on how ethical principles should be applied to the evaluation of clinical AI.

Current guidance around ethics of AI in health care commonly emphasizes accountability, transparency, bias mitigation, and governance. For example, the World Health Organization’s AI for Health 2024 guidance frames AI ethics in terms of design, deployment, use, and governance, and the International Medical Device Regulators Forum has guiding 2025 principles for good machine learning practice, which include transparency [11,12]. At a national level, the FDA has guidance from 2025 centering around transparency and risk-based evaluation of intended use of AI-based medical devices [13]. However, a key gap remains in that existing guidance broadly promotes responsible development but provides less direction for how clinical trials of autonomous AI should be evaluated as human subjects research.

Clinical research has long been guided by 7 core ethical principles delineated by Emanuel et al and endorsed by the National Institutes of Health (NIH): social and clinical value, scientific validity, fair participant selection, favorable risk-benefit ratio, independent review, informed consent, and respect for human participants [14,15]. These ethical research principles have been described as “universal” and are to be adapted to the “health, economic, cultural, and technological conditions” in which research is conducted [14]. These principles promote ethical research and guide how the scientific and social benefit of research is evaluated. Certain features of AI tools add complexity to applying these principles: the impact on multiple stakeholders (eg, developers, clinicians, and patients); the impact of deployment in different settings or populations on performance; and/or the need to account for stakeholder values throughout the process of design to deployment of AI in health care. Ethical dilemmas are likely to emerge at these tension points, where there are perceived trade-offs between different values and goals [16]. For example, a study of an AI-based chest x-ray prediction model that underdiagnosed specific subpopulations (eg, Black, Hispanic, and Medicaid subpopulations) demonstrated that there can be trade-offs between how an algorithm may acceptably account for certain demographic characteristics (eg, sex, race, ethnicity, and insurance status) versus safety for patients in general [17]. This kind of trade-off creates challenges for applying the NIH principles.

To ground these issues in a concrete case, this Delphi study used an autonomous AI screening tool for diabetic retinopathy (DR) as an exemplar. DR is a leading cause of blindness worldwide [18]. In 2018, the FDA granted the first De Novo authorization for an autonomous AI tool, which was for screening for DR, and it was then implemented in youth populations [19-21]. This case provided expert panelists with a specific example of an autonomous AI-based screening tool that has undergone FDA review and was evaluated in a clinical context.

The aim of this Delphi study was to identify areas of expert consensus regarding how the 7 NIH ethical principles should apply to clinical trials of autonomous AI tools. Further characterization of how to conduct ethical clinical research with AI tools is urgently needed to support safe implementation.

## Methods

### Overview

**Figure 1.**
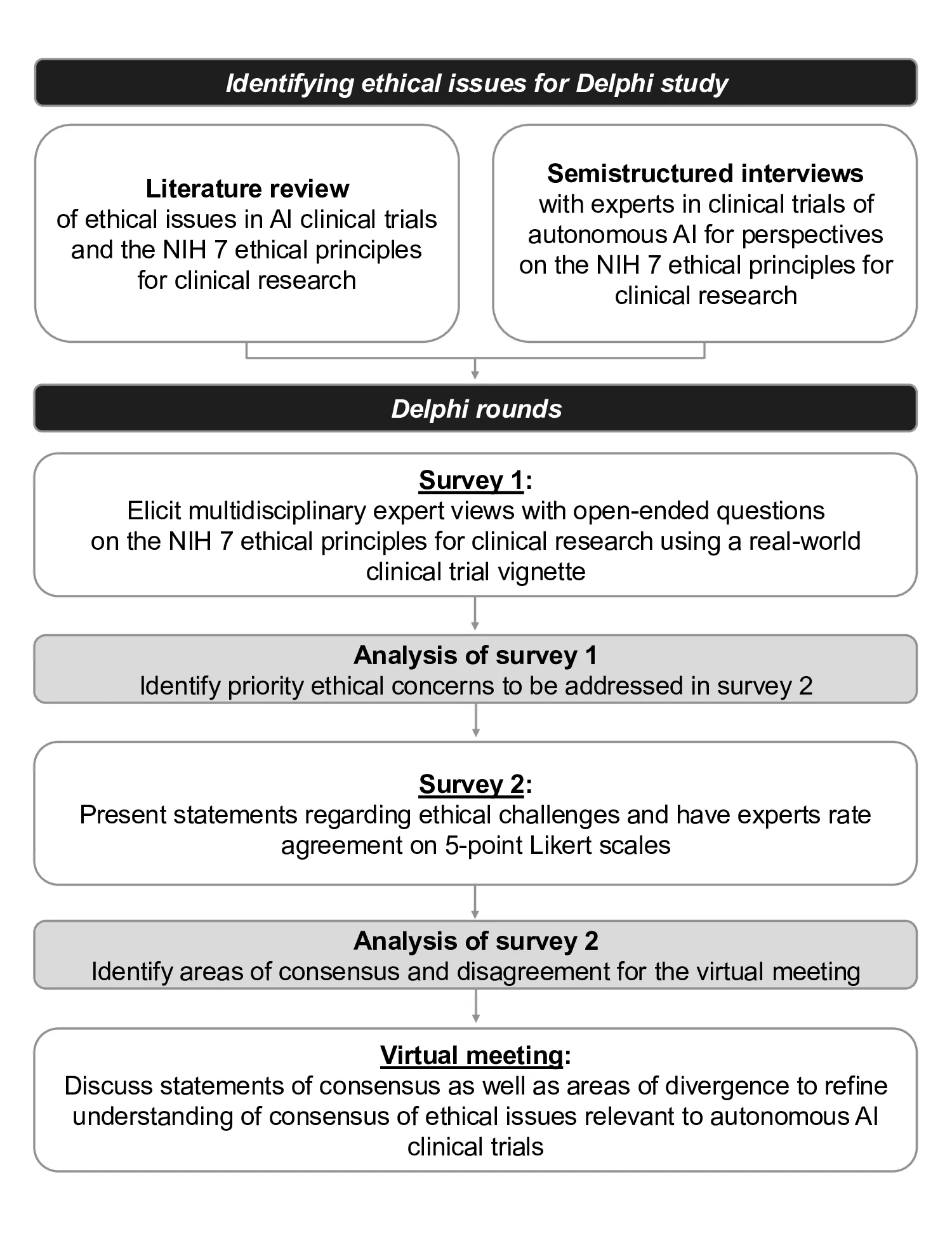
Delphi process. NIH: National Institutes of Health.

We convened a Delphi panel to understand the diverse experts’ views about the ethical considerations arising with a clinical trial of an autonomous AI that screened for DR in pediatric patients. The Delphi methodology was used because it is an established, structured, and iterative process that involves a panel of relevant experts to identify areas of consensus and dissent regarding an issue [22-24]. We used a modified Delphi approach, conducted in 3 rounds (Figure 1): a first-round survey; a second-round survey incorporating summary results from the first round; and a final virtual meeting round in which the expert panel reviewed consensus statements and further developed recommendations. See Checklist 1 for the DELPHISTAR reporting guidelines checklist.

### Composition of the Delphi Panel

Delphi panels can range in size from 10 participants to larger groups. We aimed for a panel of 12 to 15 participants. Delphi studies in the health sciences involve an average of 15 participants [25,26]. This number is thought to balance the needed range of expertise and a panel size suitable for engaged conversation for the final round meeting [24]. We identified potential Delphi participants by searching the biomedical literature in the PubMed database to identify experts in areas relevant to questions of AI use in retinopathy, including in AI and data science, ophthalmology, public health policy, law, bioethics, and patient advocacy (Table 1). Several potential participants were selected for each category of expertise and ranked based on factors such as years of experience, previous work on similar projects, and publications. Email invitations were sent based on the rankings to fill each relevant area of expertise. Twenty-four invitations were sent out in total. Ten potential participants did not respond or declined, citing time constraints. Fourteen experts agreed to participate and represented the necessary categories of expertise. Several participants had overlapping relevant areas of expertise. For example, 2 of the 3 individuals with bioethics expertise also represented expertise in AI and clinical ophthalmology. Participants were given information regarding the Delphi study and consented to participate in the study. Key considerations when selecting panel size include ensuring a diverse range of expert perspectives, timing in administration of iterative rounds of surveys, and coordination of scheduling a final group discussion with multiple experts.

**Table 1.** Characteristics of Delphi participants.

Characteristics	Values (N=14), n (%)
Sex	
Male	7 (50)
Female	7 (50)
Expertise	
Clinical trial research	1 (7)
AI and informatics	2 (14)
Clinical ophthalmology	3 (21)
Bioethics (clinical AI ethics)	3 (21)
Health policy	2 (14)
Patient advocacy	2 (14)
Industry AI	1 (7)

### Survey Stages

#### Overview

Two online structured surveys were administered via Qualtrics (Qualtrics, LLC). Participant responses were kept anonymous during the survey rounds to promote candid responses from all participants. Respondents were allowed approximately 1 month to respond, with a reminder email sent 1 to 2 weeks before each deadline to all who accepted the invitation to participate.

#### Survey 1

The first survey was initiated by informing participants that the purpose of the survey was to characterize the ethical issues relevant to the use of AI for clinical screening tools and to inform recommendations for research and policy stakeholders. The first survey elicited participants’ views on the NIH principles for ethical clinical research [14,15]. A vignette of the first NIH trial of an autonomous AI tool for DR screening was used (Textbox 1). The tool served as an exemplar case for expert panelists to consider in the first-round survey, given that it could ground the discussion in a concrete example of autonomous AI for a clinical screening application that had already undergone FDA review. Open-ended questions were used to capture participants’ views on each of the NIH principles in relation to the clinical trial vignette and to identify conflicts, if any, that potentially arose between value considerations (Table 2). The research team analyzed the responses for themes grounded in the NIH principles. We selected representative statements from the responses that addressed potential conflicts or tensions between the ethical principles, such as the tension between scientific benefit and cost-effectiveness. These statements were then used to develop the second survey.

Textbox 1. **First survey vignette.**

In 2018, the FDA approved the first autonomous AI software that interprets retinal images taken with a non-mydriatic fundus camera, providing an immediate result for diabetic retinopathy (DR) screening at the point of care (POC) for adults with diabetes. The PIs of the trial were the first to implement this technology in pediatrics, demonstrating safety, effectiveness, and equity, and cost-savings to the patient. They also found that minority youth, those with lower household income, and those with Medicaid insurance were less likely to undergo recommended screening, yet were more likely to have DR. The current trial hypothesizes that implementing POC autonomous AI in the diabetes care setting will increase DR screening rates in youth with diabetes, mitigate disparities in access to screening, and be cost-effective to the health care system. A randomized control trial conducted at two clinic sites is meant to determine 1) if autonomous AI increases screening compared to an eye-care professional (ECP), and 2) if those who screen positive by AI are more likely to go for follow-up at the ECP. There is also a prospective observational trial of this AI screening tool to determine if it mitigates disparities in screening and improves the proportion of at-risk, minority, and low-income youth who go for follow-up if their AI screen is positive. If the AI tool is shown to increase screening rates while mitigating disparities in access to care, it has the potential to reshape screening methods now and in the future.

**Table 2.** Survey 1 questions and response trends with example quotes.

Question	Trends in responses	Exemplar quotations
Question 1: clinical value Do you think there are challenges to identifying and assessing clinical value in this context? Please identify. While AI can improve access to screening for marginalized populations, it may not provide screening comparable to having access to an ophthalmologist. How, if at all, do you think this issue impacts assessment of the clinical value of the tool?	Several participants stressed the importance of determining the performance of the AI tool relative to screening by an ophthalmologist to clarify if potential gains provided by increasing screening access outweighed potential reduction in diagnostic accuracy. A few thought that AI tool performance did not need to be comparable to an ophthalmologist to have clinical value. Several highlighted the issue of the need for clinical follow-up and moral imperative to ensure that those flagged as positive in screening sought appropriate care afterward.	“Clinical value of the tool is not impacted as long as it is clearly shared by the PCP^a^ to the patient that this is not comparable screening, but rather a preliminary assessment.” “Look at algorithmic performance etc. in comparison with manual workflows...” “Value arises from what we do in response to the AI tools output...” “I think there is a debatable question about whether it’s OK to just sit back and watch how many of the kids who screen positive on AI actually follow up with an ophthalmologist, or whether because this is research, the team has an obligation to monitor who gets appointments on their own and then for those who don’t do that within a reasonable period of time, to intervene and make appointments for them.”
Question 2: social value Social value is the idea that the research question should contribute to scientific understanding of health or improve our ways of preventing, treating, or caring for people with a given disease to justify exposing participants to the risk and burden of research. What do you consider to be the social value of the AI tool in the vignette? What, if any, approaches do you think can be used to evaluate or address outcomes for that social value?	Most participants broadly agreed that the social value of the tool was its potential to improve the likelihood that people receive a timely and accurate diagnosis with the potential to narrow disparities for underserved patients. Some suggestions of approaches were the creation of a data registry for participating sites; sharing of information on AI screening at sites; and patient or community surveys and/or focus groups.	“That it could increase diagnosis of DR^b^ and reduce disparities in DR. I think that quantifying differences in diagnosis across social groups could address the social value of the intervention.” “The AI tool per se has zero social value. The social value is created from increasing screening rates while mitigating disparities in access to care.”
Question 3: potential conflicts Describe conflicts, if any, that you think could arise between clinical, social, and/or organizational values during development and/or evaluation of a diabetic retinopathy AI screening tool (eg, cost-effectiveness vs. improving care or access)? What, if any, are ways to address these conflicts? How would you ethically test cost-benefit of AI implementation? Cost can influence test parameters (such as accepting a lower sensitivity & specificity for a lower cost). - what, if any, impact do you think this has on ethical considerations?	Many participants identified potential conflicts thought not to be unique to the use of AI tools, eg, tension between high-sensitivity tests and cost burden. Several suggested conflicts specific to AI use, including issues of patient and medical professional trust in the use of AI. Transparency with patients and physicians was thought to be important if cost considerations dictate lower test sensitivity over diagnosis. Expressed concern over reinforcing “tiers of care,” with higher access to in-person testing for better-resourced groups and less-resourced groups having only AI screening.	*“...organizations should analyze all streams of costs and benefits and have a multidisciplinary body that assesses prospective AI tool deployments, following criteria that have been agreed up on in advance, which [let’s be honest] will likely include financial impact on the organization. b. I don’t see distinctive issues in performing cost-benefit or cost-effectiveness analyses for AI, compared to other clinical interventions... c. Organizations need to have total transparency with the physicians who use their tools, and potentially also with patients (depending on the tool), about what is known about model sensitivity and specificity...”* *“The main conflict here is that these efforts need to be sustainable -- and the most under-resourced areas are least likely to be able to afford additional screening, follow-up, and treatment.”*
Question 4: risk-benefit considerations In the context of the vignette, what challenges do you see in establishing an appropriate risk-benefit ratio when the risks of AI, or introducing AI into workflows, are not yet fully known? What concerns come up when applying risk-benefit considerations for this AI tool to different populations? What, if any, are ways to address these challenges?	Several participants raised the issue that there are multiple unknowns regarding introducing AI into workflows, making a risk-benefit analysis difficult. Multiple experts mentioned the risks of automation bias and algorithmic bias where risks may vary across different groups and individuals. Several highlighted difficulties of collecting data on follow-up visits, rates of DR development, acceptance of AI screening etc across different health care systems because of data compatibility and extraction.	*“One of the major reasons that clinical studies are so important is because the risks are not yet known. There are risks of automation bias and automation neglect. The importance of the trial protocol having clear specifications around how the outputs are used by the treating team is important to parsing out the effect of the model outputs specifically vs a general effect of being in a research trial [the greatest cause of bias in observational trials] The risks are also differentially distributed across populations, which we know well at this point [eg, algorithmic bias]. There is no consideration of bias in current regulatory schemes nor do most oversight frameworks consider the effects of fairness beyond the technical performance of the system alone, which is insufficient for addressing fairness in a clinical research context.”*
Question 5: equipoise Establishing equipoise can be challenging for AI medical tools because trials have to consider not just if the AI is better than current approaches but also the context into which the AI is to be applied. For an AI tool to screen for diabetic retinopathy, what do you think are important considerations for addressing equipoise?	Most participants thought the equipoise issue was not that different from that in other medical interventions. Several mentioned that equipoise should be established, not relative to the AI screening, but to the post-screening steps such as seeking appropriate follow-up testing and care; however, 2 commented that equipoise could be considered based on screening alone. Several mentioned the need to match the AI training population data to the target setting and patient population.	*“I don’t know if I agree that establishing equipoise is challenging for AI tools specifically, I think there’s not really that much different to other interventions in medicine where both of those consideration apply.”* *“...perhaps considering clinical outcomes and therapeutic interventions rather than diagnostic screening rates alone.”* *“This is important, but not the issue with screening. It seems problematic to see diagnosis of treatable illnesses as an issue because folks won’t get treatment. That seems a separate issue. I think problematic to consider this given it could lead to not wanting to screen among uninsured folks for example.”*
Question 6: access to therapeutic interventions AI screening of populations who have difficulty accessing health care may not improve their access to therapeutic interventions, even if it does improve diagnostic screening. How do you think this impacts ethical issues at play in the vignette?	There were divided opinions over issues of AI that increased rates of diagnosis but unaccompanied by appropriate follow-up and therapeutic intervention. Some expressed concern over distribution of resources and investment if patients were not ultimately helped. Others still saw value in improving diagnostic screening even if follow-ups were not impacted.	*“We will never know disparities in treatment exist if we don’t diagnose as many people as possible -- as long as it is something that is meaningful to diagnose.”* *“This is an uncertainty that should be acknowledged. We can draw from lessons learned from other screening or diagnostic programs. In melanoma for example, there is a data to show increased rates of diagnosis does not improve outcomes in the long-term - therefore the impact on resources, cost, patient distress are all consequences of an intervention that may not be helpful.”* *“It shows how technology is always only one piece of a larger problem. The ethical issue then is insufficient scientific practices contribute to research waste...”*
Question 7: potential impact of existing health inequities What concerns, if any, would you have for the above scenario regarding how existing health inequities might impact ethical application of the AI tool?	A majority of the participants had concerns regarding the impact of existing health inequities on the AI tool application. Opinions were divided over whether this concern was different relative to non-AI health care interventions.	“This is not much different to current standard in health care and research. The best approach is to consider inclusivity in research design to encourage typically under-represented groups to participate in the trial. If certain groups are less likely to go for screening, you still won’t be reaching them just because you added an AI tool to a place where they are already less likely to go. And if you don’t change the human aspects of healthcare (eg, anti-racism, inclusive care) then it doesn’t matter how good the technology actually is, it won’t improve things.” “Access is a major problem for implementation - will the tool, if built upon research in this population, make its way back for use by this population?”

a PCP: primary care provider.

b DR: diabetic retinopathy.

#### Survey 2

The purpose of the second survey was to further develop an understanding of the issues relevant to AI clinical trials and the 7 principles for ethical research that the participants thought should be prioritized. Participants were asked to rate the representative statements according to the level of agreement with the statement on a Likert scale of 1 to 5 (1 being least and 5 being most; Textbox 2). In accordance with Delphi methodology, statements of consensus (at least 80% rating agreement) and moderate agreement (60%‐80% rating agreement) were identified, along with certain particularly divisive statements (with no more than one neutral rating and otherwise evenly split across the agree and disagree ratings). Responses were descriptively analyzed by the research team, and an initial summary of consensus recommendations was then drafted for the final round. We used Delphi standard cutoffs regarding agreement [27,28].

Textbox 2. **Survey 2 statements of consensus, moderate agreement, and divisive statements.**

Strong agreement

- To evaluate the clinical value of the diabetic retinopathy AI tool, there first needs to be a comparison to the quality of care that would be received when an ophthalmologist does the task.
- The social value of an AI tool should be measured by examining the relative gains or losses between different populations, such as the number of people getting diagnosed and people actually getting treatment.

Risk-benefit ratio

- The clinical trials themselves establish the risks involved, and it will be important, as part of that, to establish how these risks are unequally distributed across different populations.
- For the evaluation of clinical AI interventions, it is particularly important to identify and share information (eg, characteristics) regarding the target population and research population.
- A key problem in clinical evaluation of AI tools is being able to generalize that the AI will be effective in different sites and different populations.
- In evaluating clinical AI, it is particularly important to ensure that there is discussion of how bias sensitivity-specificity is set for majority versus minority populations.
- As existing inequities will likely impact any potential benefits of the AI tool for specific populations, there needs to be initial investment in understanding those inequities before approving use of AI tools in different populations.
- For consent purposes, it is important that patients understand that their data are being used for clinical trials as well as potentially for other uses, such as developing pharmaceuticals.
- There need to be more clearly delineated avenues for patients to contribute their perspectives to the process of evaluating clinical AI tools.

Moderate agreement

- There is a need for a new ethical framework that does more to anticipate downstream implications or social value of the AI tool being clinically evaluated.
- Social value for an AI tool cannot be evaluated as a stand-alone issue because the social value comes from the related systemic support factors, such as having structural factors in place to increase screening, diagnosis, and treatment overall.

Divisive statements

- Evaluating a clinical AI intervention is not significantly different from evaluating any other type of clinical intervention.
- Evaluating clinical AI is different from evaluating other types of clinical interventions because the outcome of an AI intervention may not be transferable to different sites or populations.
- Evaluating clinical AI is different from evaluating other clinical interventions because the outcome of an AI intervention is often more dependent on systemic factors, such as access to additional care afterward.
- Evaluation of the cost-effectiveness of an AI tool needs to be broken down according to different populations.

#### Final Virtual Meeting

The final round of the Delphi study took place as a virtual meeting using Zoom (Zoom Communications, Inc) for group discussion. There was a structured discussion in which the panelists reviewed the results of survey 2, focusing the discussion on the statements for which there was consensus, as well as the statements that were most polarized between strongly agree and strongly disagree in the responses. The discussion also allowed panelists to clarify some of the reasoning behind the statements, as well as to endorse revisions to the statements as a group. In this way, the meeting served to finalize the recommendations by the expert panel.

### Ethical Considerations

The protocol for this study was approved by Stanford’s Institutional Review Board (Protocol 69318). Informed consent was obtained, with a waiver of documentation approved by the institutional review board. Data from surveys 1 and 2 were anonymized. No compensation was used.

## Results

### Survey 1

Of the 14 experts who agreed to participate in the panel, 12 (85.7%) responded to survey 1. The questions and exemplar quotes from survey 1 respondents are presented in Table 2.

Participant responses regarding the ethical principle of “Clinical and Social Value” indicated several key tensions in applying this principle to the evaluation of an autonomous AI tool. For example, several participants identified the need for ensuring clinical follow-up as a potential challenge to substantiating the clinical value of an AI tool. Most participants broadly saw potential social value, that is, that research should contribute to scientific understanding of health or improve ways of preventing, treating, or caring for people with a given disease to justify participants’ exposure to the risk and burden of research. The social value was reported as the potential to provide a more timely and accurate diagnosis for a disease with treatment available for mitigating further disease progression and to narrow the gap in access to screening for underserved populations. Participants also provided several suggestions for how to evaluate or address social value further, which included community focus groups and a data registry for participating sites.

Most participants raised cost considerations as potentially conflicting with the clinical or social value of the AI tool. At the same time, most participants felt that the cost-benefit analysis of autonomous AI is no different than the assessment of non-AI health care interventions. However, some responses reported that a concern specific to AI tools in health care was the potential impact of patient, public, and providers’ trust in an AI tool on the clinical impact of the tool.

Regarding the principle of “Favorable Risk-Benefit Ratio,” the risk-benefit considerations of autonomous AI were seen to be multifactorial with variable unknowns regarding the introduction of AI into workflows and barriers to data extraction and collection, making risk-benefit analysis difficult. Participants also broadly reported that the consideration of clinical equipoise for AI-based clinical trials was similar to those of non-AI clinical trials.

The issue of health disparities was seen in survey responses regarding several of the principles for ethical research, such as “Clinical and Social Value,” “Fair Subject Selection,” and “Respect for Enrolled Subjects.” The majority of participants had concerns regarding the impact of existing disparities in access to care on the AI tool application development and bias. Participant responses varied on the relevance of access to diagnostic screening without sufficiently addressing barriers to access to therapeutic care. Most participants emphasized that this was not a new issue in clinical research. A few participants posited autonomous AI clinical trials as potential research waste, where resources could be allocated elsewhere to improve care access. Some participants saw access to screening as beneficial, with one stating that screened patients would still have the knowledge of the presence of a health irregularity and may access care down the road.

### Survey 2

Of the 14 experts who agreed to participate in the panel, 10 (71.4%) responded to survey 2. Two experts who missed the survey deadline emailed written comments on the survey questions that were incorporated into the survey 2 findings. Textbox 2 summarizes statements of consensus (at least 80% rating agreement), moderate agreement (60%‐80% rating agreement), and divisive statements (with no more than one neutral rating and otherwise panelists evenly split across agree and disagree ratings).

### Final Round Virtual Meeting

The final round meeting had both synchronous and asynchronous participation by 13 (92.9%) of 14 participants. Panelists identified reducing bias, improving access to care, ensuring downstream access to recommended treatments, promoting transparency regarding the representativeness of the training data, determining prespecified outcomes with respect to the reference standard, having clear pretrial discussion of cost or cost savings, and engaging relevant stakeholders (eg, clinicians and patients) as central priorities. A broader theme derived from the discussion was a tension that although many ethical concerns in AI trials resemble longstanding issues in clinical research, autonomous AI trials may provide an opportunity to address these issues more systematically. The following subsections summarize the combined results.

### Opportunities to Advance Ethical Research Principles

Participating panelists emphasized that AI tools are distinct from non-AI medical interventions, yet the issues faced in conducting clinical trials are similar. Panelists who expressed the position that AI tools did not present significant differences for clinical trials tended to point out that priority ethical issues for AI, such as bias or informed consent, have also, historically, presented problems for traditional medical interventions. Given the current investment in AI devices, panelists saw there being a potentially useful moment for AI developers to reflect on bias and avoid the development of medical devices that would unintentionally amplify those inequalities. Some panelists pointed toward ways that they saw AI presenting distinct ethical challenges, including clinicians’ limited understanding of potential AI tool limitations and the need to ensure context-specific usability within diverse clinical workflows. Panelists agreed on the existence, but not the magnitude, of these differences and on whether such differences warrant adjustments to the ethical framework for clinical trials. Regardless of the distinctiveness of AI evaluation, expert panelists agreed that efforts to improve clinical trials of AI could also provide an important opportunity to address longstanding ethical issues of bias, informed consent, and access to care.

### Clinical Value

The expert panel highlighted the need for trials to prespecify outcomes with respect to the reference standard and to describe any challenges in comparison, such as situations in which the current professional medical examination screens for multiple conditions simultaneously, while the AI tool screens for only one condition. Panelists frequently used the term “trial design” broadly to include aspects such as randomization strategy, comparator arm selection, and primary endpoint definitions.

Several of the panelists raised questions of how the autonomous AI tool for DR screening should be compared to screening by an ophthalmologist. Panelists were interested in this comparison as part of considering the broader issue of whether reliance on AI for DR screening as a cost-effective tool in low-resource settings could create or reinforce a lower tier of care. In the vignette considered by the panel, the AI tool had been evaluated against a level 1 reference standard based on clinical outcome, indicating that the AI for DR screening tool was more accurate than a human clinician at screening for DR, as this was raised by one of the panelists. Nevertheless, panelists agreed that trial reports should clearly explain the chosen reference standard and how it relates to the clinical care patients would otherwise receive.

### Bias and Stakeholder Engagement

The panelists endorsed several approaches that are meant to address the use of AI across different contexts and for populations that may not be sufficiently represented in training datasets. The clinical trial of an AI tool for DR screening demonstrated that, when placed at point-of-care sites, the tool could improve screening rates among populations historically lacking access, and its performance did not differ across minoritized subgroup populations. However, panelists raised concerns more broadly that a lack of sufficient representation of minoritized populations in training data can lead to systematic bias that undermines the potential benefits of increased screening. There was general agreement among the panelists of the importance of transparency and sharing of information regarding the demographics of training datasets.

The impact of context and integration into workflow on the effectiveness and accuracy of AI tools was highlighted as a particularly challenging issue for evaluation. Panelists supported practices of stakeholder engagement for the development and implementation of AI tools in health care. Panelists noted, for example, engaging clinicians regarding practical aspects of how the AI tool might fit into their workflow could also identify the way that use of the tool in those different contexts could support or create challenges for effective use. One concern raised was that a tool that only evaluates for one condition may not be as useful as a tool that can evaluate several potential eye conditions.

Several panelists discussed how community and/or patient engagement could also be used to help identify factors that impact the usefulness of AI tools for specific purposes and that these kinds of engagement could help identify systemic factors that affect bias and fairness. Panelists agreed that the specific stakeholders needing to be engaged might vary according to the type of tool and purpose. Panelists noted that stakeholder engagement has become more common in recent years for clinical research, and they recognized the need to include such engagement with AI to support these kinds of efforts regularly as part of the process of development and implementation.

### Cost-Effectiveness

Several panelists expressed concern that, generally speaking, AI could make it easier to implement different tiers of care, where the price point of an AI tool could be used to lower costs as well as quality of care for lower-resourced patients. While that was not as likely the case with the specific AI tool for DR screening, given studies found that the tool performed better than clinicians on average, panelists wanted to note this issue for the field in general. At the same time, panelists acknowledged that such concerns regarding cost are not necessarily unique to AI tools within health care. Several panelists suggested that cost savings with an AI tool could present an opportunity within a health care institution or system in ways that benefited underserved patients, such as increasing support for additional access to treatment.

### Downstream Implications of the AI Tool

Panelists gave substantial attention to the downstream implications of the AI tool for DR screening and whether evaluation of the tool should account for access to treatment options after a positive screen. Several panelists expressed ethical concerns over an AI tool that might increase the number of people identified as potentially having DR but then leave those people without a way to address their condition. Participants noted that this concern of a diagnosis without access to treatment is certainly not limited to AI tools and that clinical trials are generally not expected to resolve system-level gaps in access. However, the majority of panelists agreed that such downstream implications should be considered when evaluating when and how to use an autonomous AI tool. A few panelists also raised a potential benefit that having an AI screening tool implemented might free up clinician time for treating rather than screening. They noted, however, that whether this benefit would be realized would still depend upon the resources and structure of the health system in which the screening takes place.

The Delphi panel concluded that to apply the 7 ethical principles of research to autonomous AI clinical trials, it is particularly important to incorporate evaluation of potential downstream implications, such as access. Panelists acknowledged that a number of the concerns that they prioritized for ethical clinical research with AI could be applied to conventional medical interventions as well, such as addressing the risk of bias and incorporating engagement with patients and other stakeholders. At the same time, however, the expert panelists overall agreed that addressing the framework for research ethics of autonomous AI clinical trials could also be seen as a greater opportunity to advocate for structural changes that promoted equity more broadly in clinical research and health care.

## Discussion

### Principal Findings

This Delphi study identified several areas of expert consensus regarding how the 7 NIH ethical research principles should be applied to clinical trials of autonomous AI. Consensus centered on practical features of trial design and reporting, including documentation of training and validation data, prespecified outcomes and reference standards, assessment of performance across populations and settings, engagement with relevant stakeholders, and consideration of cost, access, and follow-up care. Although panelists differed on the extent to which they viewed autonomous AI as distinct from conventional medical interventions, they agreed that many concerns raised by autonomous AI tools reflected longstanding challenges in clinical research. At the same time, panelists viewed the current development of autonomous AI tools as an opportunity to address such concerns more systematically within clinical trial design and evaluation. Overall, these findings suggest that applying established research ethics principles to autonomous AI clinical trials requires evaluating tool performance, while also considering downstream effects on patients, clinicians, and health systems.

These findings extend existing applications of the NIH research ethics principles by specifying how clinical value, scientific validity, and equity should be considered in autonomous AI clinical trials. The panel’s emphasis on downstream implications aligns with prior work emphasizing that ethical evaluation of medical AI depends not only on model performance but also on implementation context and real-world deployment [3,7,29]. This focus is also consistent with qualitative evidence showing that clinical AI implementation is shaped by interdependent factors across multiple stakeholder groups [30] and that developers can recognize potential AI-related harms to patients, groups, and health systems though they vary in their level of perceived responsibility in mitigating harms [31]. The panel’s prioritization of bias and representativeness is further supported by evidence that addressing AI bias requires both technical and social approaches [29,32], that most FDA-evaluated AI tools do not report demographic characteristics of training data [33], and that most AI clinical trials internationally are single-center studies [34]. Qualitative work on AI health datasets also supports the importance of documenting data representativeness and intended use in terms of societal impact [35]. Taken together, our Delphi study and the existing literature support incorporating attention to representativeness, subgroup performance, downstream access, and lifecycle costs into the ethical evaluation of autonomous AI clinical trials, particularly for tools intended for low-resource settings.

Our earlier qualitative study of clinical trials involving AI for DR screening identified a range of ethical challenges that were not fully addressed by existing research ethics frameworks, including issues related to social value, scientific validity, and informed consent [21]. However, the participants in that study were restricted to investigators involved in AI clinical trials for the specific purpose of screening for DR. By engaging a broader, multidisciplinary panel of experts, including experts in health policy and patient advocacy, panelists were able to reach consensus on several key overarching issues affecting the application of ethical research principles to AI clinical trials. The study resulted in the development of 9 key actionable recommendations (Table 3). Implementation of these actions would help regulators and investigators promote equitable potential benefits and reduce potential unintended consequences of autonomous AI clinical trials.

**Table 3.** Application of ethical considerations in real-world clinical AI trial settings.

Action items	Description
Increase transparency of AI training and validation data	Trial protocols should describe how the training data compare with the intended trial population and identify any representativeness gaps before enrollment begins.
Assess bias before deployment	The statistical analysis plan should include prespecified subgroup analyses and define what level of performance difference would require modification, monitoring, or nondeployment.
Evaluate performance across clinical settings	Trials should include sites that reflect the settings where the AI tool is likely to be used and assess whether performance changes across care environments.
Address health inequities before implementation	Investigators should identify structural barriers that may affect AI performance or patient outcomes and describe mitigation steps before widespread use.
Incorporate stakeholder engagement	Trial development should document how input from affected patients, clinicians, or community representatives shaped study design and implementation.
Strengthen informed consent practices	Consent materials should explain the role of the AI tool in care decisions and describe whether patient data may be used beyond the immediate trial.
Compare AI tools with existing standards of care	AI tools should be evaluated against current clinical practice, including effects on diagnostic performance and patient-relevant outcomes.
Evaluate downstream care access	Trials should assess whether patients can obtain appropriate follow-up care after AI-generated recommendations, rather than measuring accuracy alone.
Assess cost and access implications	Trial designs should take into account whether AI implementation could shift costs to patients, clinics, or under-resourced health systems.

### Limitations

The study has several limitations. While the number of experts on the Delphi panel was within the recommended range for Delphi practices and was highly multidisciplinary, the panel may not represent the full range of perspectives. In addition, all expert panelists were based in the United States, which could limit the international applicability of the recommendations in terms of familiarity with other practice and regulatory contexts. As a Delphi study, the approach is based on the idea that structured discussion and review of the research questions by relevant experts yields practical guidance. Additionally, the regulatory and technological landscape for autonomous AI is rapidly evolving, and therefore, these recommendations may require ongoing refinement as new tools emerge. This study also did not directly address environmental sustainability associated with AI development and monitoring, which could be an area of future consideration.

### Conclusions

Autonomous AI clinical trials raise ethical considerations that extend beyond technical accuracy, particularly when tools are intended for use across diverse populations and health care settings. The recommendations developed through this Delphi process suggest that ethical evaluation of these trials should account for the provenance and representativeness of training and validation data, the possibility of biased performance, stakeholder perspectives, access to follow-up care, and cost-related effects. As autonomous AI tools continue to be tested in clinical research, these considerations can help shape regulatory and policy expectations for the ethical development and evaluation of autonomous AI clinical trials.

## Supplementary material

10.2196/91472 Checklist 1 DELPHISTAR reporting guidelines checklist.

## Abbreviations

DR: diabetic retinopathy
FDA: US Food and Drug Administration
NIH: National Institutes of Health
