# Supplementary material for: Ethics of Autonomous AI Clinical Trials: Delphi Study
Source: J Med Internet Res. 2026 Aug 21;28:e91472. doi: 10.2196/91472 (PMC13496388; doi:10.2196/91472)
Supplement: Supplementary file 1 [file jmir-v28-e91472-s001.docx]

**Delphi studies in social and health sciences – recommendations for an interdisciplinary standardized reporting (DELPHISTAR).**

From: Niederberger, M., Schifano, J., Deckert, S., Hirt, J., Homberg, A., Köberich, S., Kuhn, R., Rommel, A., Sonnberger, M. & the DEWISS network (2024). Delphi studies in social and health sciences—Recommendations for an interdisciplinary standardized reporting (DELPHISTAR). Results of a Delphi study. *PLoS ONE 19(8):* e0304651. <https://doi.org/10.1371/journal.pone.0304651>

More information under OSF ([https://osf.io/gc4jk)](https://osf.io/gc4jk) and DEWISS ([https://delphi.ph-gmuend.de/)](https://delphi.ph-gmuend.de/)

**What is the aim of DELPHISTAR?**

- Improve, harmonize, and make the reporting in publications on Delphi studies comparable
- Facilitate the evaluation of Delphi studies including during peer review processes
- Reduce, and ideally prevent, inconsistencies and unclear descriptions in publications on Delphi studies
- Raise awareness of the diversity among the Delphi variants and of their specific potentials and challenges

**DELPHISTAR is a Delphi reporting guideline that is:**

- valid for all Delphi variants (e.g., classic Delphi, real-time Delphi, group Delphi, policy Delphi, argumentative Delphi, café Delphi)
- applicable to different purposes (e.g., Delphi studies to establish consensus, to gather expert judgments or to forecast)
- given equal consideration in the health and social sciences

**This reporting guideline is meant for studies using Delphi techniques in the health and social sciences.** These also include all Delphi variants and modifications that meet the following criteria:

1. Survey of several people (=experts) with specialized knowledge (e.g., operational knowledge, experiential knowledge, functional knowledge, contextual knowledge)
2. Structured communication process
3. Carrying out at least two survey rounds or the option to respond at least two times
4. Feedback: the (interim) results are presented to the experts starting from the second round
5. Basis is a quantitative questionnaire with the possibility to contribute or supplement arguments for the respective position
6. Quantitative and qualitative answer are systematically analyzed (quantitative: e.g., descriptive statistics, qualitative: e.g., thematic analysis)

This reporting guideline is available in English and German a[t https://delphi.ph-gmuend.de/activities/delphistar (](https://delphi.ph-gmuend.de/activities/delphistar)last update October 2024).

**Contact**

Prof. Dr. Marlen Niederberger

E-mail: marlen.niederberger@ph-gmuend.de

Department of Research Methods in Health Promotion and Prevention

Institute for Health Sciences, University of Education Schwäbisch Gmünd,

Oberbettringer Straße 200, 73525 Schwäbisch Gmünd, Germany

| **Topic** | **Section** | **Item** | **Checklist Item** | **Location where**  **item is reported** | **Exemplary wording** |
| --- | --- | --- | --- | --- | --- |
| **I**  **Title and**  **Abstract** |  | 1 | Identification as a Delphi study in the title | In the Title (p.1). | What is a public health intervention? Results of a Delphi study. |
|  |  | 2 | Identification as a Delphi study in the abstract | In the Abstract (p.2). | A Delphi study was selected to answer the research question. |
|  |  | 3 | Structured abstract | In the Abstract (p.2). | e.g., background, method, results and discussion |
| **II**  **Context** | **Formal** | 4 | Information about the sources of funding | In the Funding section (p.22). | The Delphi study was funded by [SOURCE]. |
|  |  | 5 | Information about the team of authors and/or researchers  (e.g., discipline, institution) | Diverse interdisciplinary team identified in the author listings representing Medicine, Ethics, Ophthalmology, and Computer Engineering (p.1). | The Delphi study was conducted by an interdisciplinary team with representatives from medicine, public health, and health promotion. |
|  |  | 6 | Information about method consulting | Authors included experts experienced in Delphi studies so no consulting in regard to the method was needed. | The study group was advised by experts from [INSTITUTION] regarding statistics.  Or:  No consulting in regard to method took place. |
|  |  | 7 | Information about the project background | Project background is presented in the Abstract and Introduction sections (p.2-5). | The Delphi study was part of a mixed methods study on [AIM]. |
|  |  | 8 | Information about the study protocol | Details of the study protocol are presented in the Methods section (p.5-9). | The study protocol is available at [LINK]. |
|  | **Content** | 9 | Justification of the chosen method (Delphi) to answer the research question | Justification for the chosen Delphi method is stated in the Methods Overview (p.5). | The Delphi method is suitable for answering the research question because it systematically gathers the judgments of different expert groups and can identity agreement and disagreement. |
|  |  | 10 | Aim of the Delphi study (e.g., consensus, forecasting) | The aim of the Delphi study is stated in the last paragraph of the Introduction section (p.5). | The aim of the Delphi study is to find consensus on criteria to define a public health intervention. |
| **III**  **Method** | **Body &**  **Integration of knowledge** | 11 | Identification and elucidation of relevant expertise, spheres of experience, and perspectives (e.g., theory, practice, affected groups, disciplines) | The choice of expert representation is stated in the Composition of the Delphi Panel subsection of the Methods section and is shown to include AI and data science, ophthalmology, public health policy, law, bioethics, and patient advocacy (p.6). | The experts represent the sciences and clinical practice because [REASON]. |
|  |  | 12 | Handling of knowledge, expertise and perspectives which are missing or have been deliberately not integrated | No relevant area of expertise was deliberately omitted or considered missing. | If it is not possible to recruit experts specialized in [AREA], this will be openly communicated to the other experts during the Delphi study. |
|  |  | 13 | Basic definition of expert^1^ | Experts were identified as authors in the relevant biomedical literature (p.6). | A person who has been active in the area for at least [NUMBER] years is considered to be an expert. |
|  | **Delphi variant and modifications** | 14 | Identification of the type of Delphi variant and potential modifications (e.g., classic Delphi, real-time Delphi, group Delphi) | Description of the modified Delphi approach conducted in three rounds is provided in the Overview subsection of the Methods Section (p.5). | A classic Delphi study was used [LITERATURE REFERENCE]. |
|  |  | 15 | Justification of the Delphi variant and modifications, including during the Delphi study, if applicable | Not applicable. | If the willingness to participate clearly decreases between the first and second round, a third round will not be held. |

| **Topic** | **Section** | **Item** | **Checklist Item** | **Location where**  **item is reported** | **Exemplary wording** |
| --- | --- | --- | --- | --- | --- |
|  | **Sample of experts** | 16 | Selection criteria for the experts (per round, per expert group if applicable) | Description of author participation in each survey round is provided in the first paragraph of Results section of the Survey 1, Survey 2 and Final Round Virtual Meeting subsections (p.9,15,16). | All of the experts who met the definition were invited to the first round. All of the experts who completed the previous round were invited to participate in the subsequent round. |
|  |  | 17 | Identification of the experts | The identification of experts based on publications in the PUBMED database is stated in the first line of the Composition of the Delphi Panel subsection of the Methods section (p.6). | The experts were identified based on publications in [DATABASE]. |
|  |  | 18 | Information about recruiting and any subsequent recruiting  of experts | Information about recruiting of experts is presented in the Composition of the Delphi Panel subsection (p.6). | The experts were informed about the Delphi study and invited to participate. |
|  | **Survey** | 19 | Elucidation of the content development for the questionnaire^2^ | Questionnaire content development is detailed in penultimate paragraph of the Introduction section, Figure 1, and Methods sub-section Survey Stages (p.5-7). | The questionnaire was developed based on the results of systematic reviews [LITERATURE REFERENCE]. |
|  |  | 20 | Description of the questionnaire (content and structure) | Description of the questionnaire is provided in Figure 1, Methods sub section Survey Stages, and Tables 3 and 4 (p.5-7, 9-16). | The questionnaire was divided into three segments on [TOPICS]. The statements made in the questionnaire were evaluated using standardized items, with the option to comment in free-text boxes. |
|  | **Delphi rounds** | 21 | Number of Delphi rounds | Number of Delphi rounds is presented in the Methods Overview sub section (p.5). | Three Delphi rounds were held. |
|  |  | 22 | Information about the aims of the individual Delphi rounds | Aims of the three rounds are presented in Methods sub section Overview and Figure 1 (p.5-6). | The first Delphi round focused on exploring relevant aspects. These aspects were then presented to the experts in the second Delphi round for standardized evaluation. |
|  |  | 23 | Disclosure and justification of the criterion for discontinuation | Methods sub section Overview indicates that the number of rounds was defined in advance to be a maximum of three rounds (p.5). | The number of rounds was defined in advance to be a maximum of three rounds. |
|  | **Feedback** | 24 | Information about what data was reported back per round | Information reported back per round is summarized in Figure 1 and detailed in the final paragraph of Methods; Survey Stages; Survey 1 and Survey 2 sub sections and Tables 3 and 4 (p.5-16). | In terms of feedback, we shared the statistical results plus the summary of the open responses. |
|  |  | 25 | Information on how the results of the previous Delphi round were fed back to the experts surveyed (e.g., via frequencies, mean values, measures of dispersion, listing of comments) | Information reported back per round is summarized in Figure 1 and detailed in the final paragraph of Methods; Survey Stages; Survey 1 and Survey 2 sub sections and Tables 3 and 4 (p.5-16). | Mean values, standard deviations and percentage frequency distributions were reported. |
|  |  | 26 | Information on whether feedback was differentiated by specific groups (e.g., by field of expertise, institutional affiliation) | Not applicable. Participants were not divided into specific groups. | The feedback was aggregated across all expert groups. |
|  |  | 27 | Information about how dissent and unclear results were handled | Results showing disagreement were presented iteratively for further evaluation in the next Delphi round (p.5-9). | The results showing dissent were presented again for evaluation in the next Delphi round. |
|  | **Data analysis** | 28 | Disclosure of the quantitative and qualitative analytical strategy | As described in the Methods Section Survey 1 sub section, open-ended questions of survey 1 were analyzed thematically, and in Survey 2 sub section, the quantitative items in Survey 2 were analyzed descriptively (p.5-9). | The quantitative items were descriptively analyzed. The open-ended items were analyzed using thematic analysis [LITERATURE REFERENCE]. |
|  |  | 29 | Definition and measurement of consensus | The definition and measurement of consensus are detailed in the Methods Section Survey 2 sub section (p.8). | Consensus was defined as percentage agreement, meaning that agreement was  assumed if at least 80% of the respondents agreed on an item. |
| **Topic** | **Section** | **Item** | **Checklist Item** | **Location where**  **item is reported** | **Exemplary wording** |
|  |  | 30 | Information on group-specific analysis or weighting of experts  (e.g., theory vs. practice, discipline-specific analysis) | Not applicable. There was no group-specific analysis or weighting of experts. | In the analysis, the mean values for percent agreement are weighted for each expert group in terms of the number of group members. |
| **IV**  **Results** | **Delphi process** | 31 | Illustration of the Delphi study (e.g., in a flow chart) | Flow chart of the Delphi study is shown in Figure 1 (p.6). | A summary of the Delphi study is illustrated in a flow chart (Figure 1). |
|  |  | 32 | Information about special aspects during the Delphi study (e.g., deviations from the  intended approach with justification) | Not applicable. | During the Delphi study the political discussion mentioned climate change and the effects on health. It is possible that this influenced the experts' responses. |
|  |  | 33 | Number of experts per round (both invited and participating) | The number of experts per round is clearly stated in the first paragraph of the Results section Survey 1, Survey 2 and Final Round Virtual Meeting sub sections (p.6,9,15-16). | The number of experts participating in the first Delphi round was [NUMBER], and the number of experts in the second round was [NUMBER]. This corresponds to a response rate of [NUMBER]% in the first round and [NUMBER]% in the second round. |
|  | **Results** | 34 | Presentation of the results for each Delphi round and the final results | Results presented by round (p.9-19). | In the first Delphi round [NUMBER]% of the experts agreed, in the second [NUMBER]%, and in the third [NUMBER]%. |
| **V Discussion** | **Quality of findings** | 35 | Highlighting the findings from the Delphi study | Results section and Table 5 highlight the actionable findings from the Delphi study (p.9-21). | The central findings can be summarized as follows: [STATE FINDINGS]. |
|  |  | 36 | Validity of the results (e.g., transferability of the findings) | The Limitations Section addresses these issues (p.21). | The results are not transferable to other countries due to different legal regulations. |
|  |  | 37 | Reliability of the results (e.g., split half, inter-rater reliability) | Open-ended responses were analyzed by multiple independent reviewers as part of research team. | The responses in the free-text comments were analyzed by two independent reviewers [SPECIFY]. |
|  |  | 38 | Reflection on potential limitations (e.g., number of experts, response bias) | Potential limitations are reflected in the Limitations Section (p.21). | The results are to be viewed critically with regard to the composition of the panel because [REASONS]. |

1 “Experts” are the participants; these can be people from academia, practice, or representatives of lived experience (e.g., patients, family members).
2 The term “questionnaire” stands for the survey instrument regardless of whether quantitative or qualitative items are integrated or weighted.
